# Supplementary material for: Targeting ADAR stability: a novel approach to combat glioblastoma
Source: Front Oncol. 2026 Aug 6;16:1827793. doi: 10.3389/fonc.2026.1827793 (PMC13489870; doi:10.3389/fonc.2026.1827793)
Supplement: Supplementary file 1 [file SupplementaryFile1.zip › Supplementary file 1/Genes_up-regulated_by_reactive_oxigen_species_(ROS).pdf]

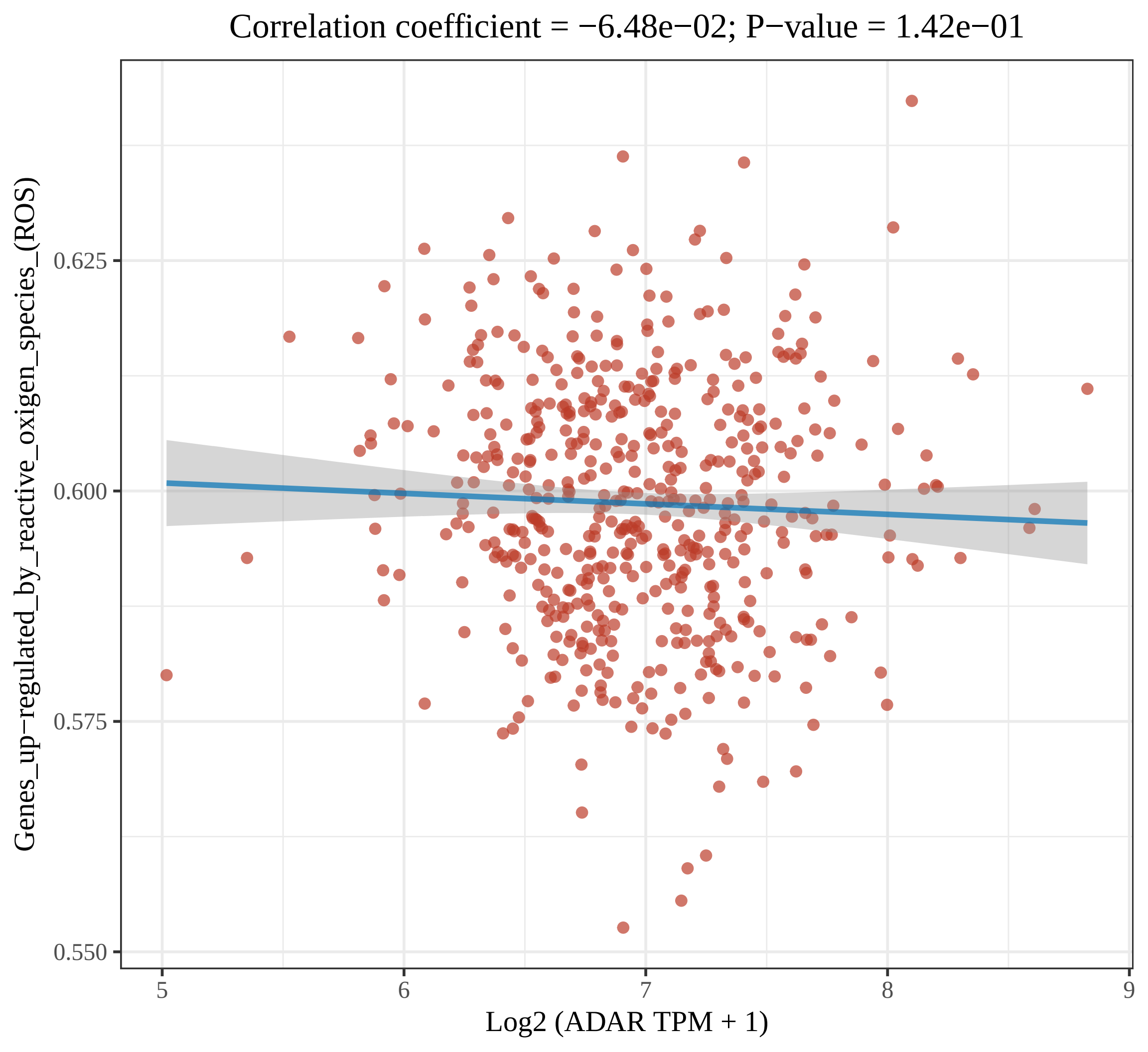
Correlation coefficient = −6.48e−02; P−value = 1.42e−01
Genes_up−regulated_by_reactive_oxigen_species_(ROS)
0.625
0.600
0.575
0.550
5
6
7
8
9
Log2 (ADAR TPM + 1)
